# Supplementary material for: OsmiRNA5488 Regulates the Development of Embryo Sacs and Targets OsARF25 in Rice (Oryza sativa L.)
Source: Int J Mol Sci. 2023 Nov 13;24(22):16240. doi: 10.3390/ijms242216240 (PMC10671434; doi:10.3390/ijms242216240)
Supplement: Supplementary file 1 [file ijms-24-16240-s001.zip › Supplementary document.pdf]

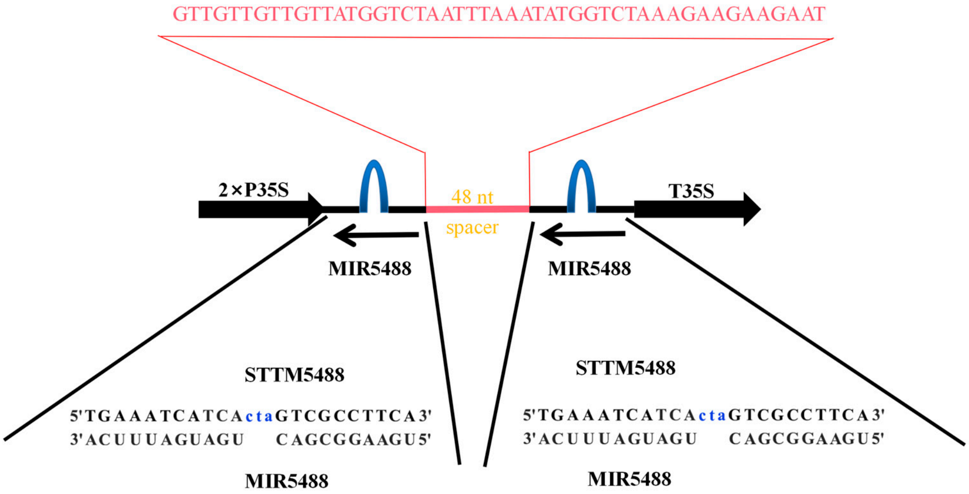

Figure S1. Diagram of STTM5488 structure showing the design strategy. Orange indicates the spacer region and the spacer sequence. Blue indicates the bulge sequences in the miRNA binding sites. Red indicates the nucleotides. nt, nucleotides.

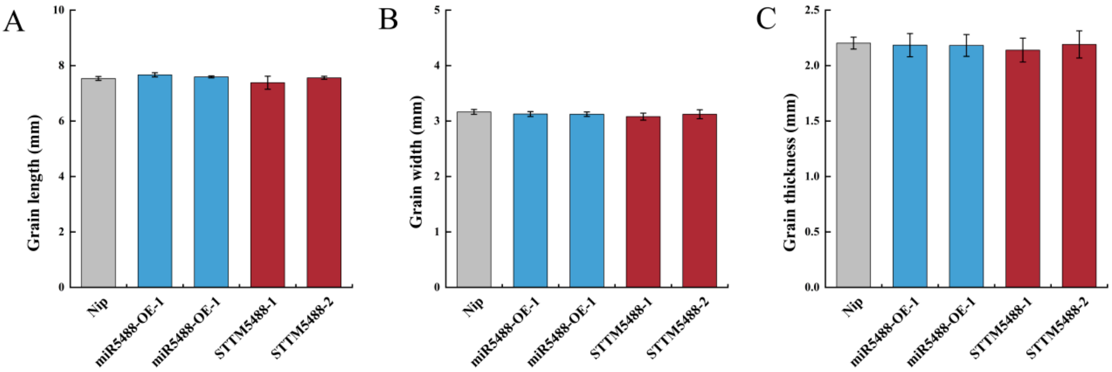

Figure S2. (A,B,C) Grain length (A), grain width (B), grain thickness (C) of Nip ,miR5488-OE and STTM5488 plants. Data are means (±s.e.m.) (n=20 plants)
